# Synthesis of Azulene Derivatives from 2*H*-Cyclohepta[*b*]furan-2-ones as Starting Materials: Their Reactivity and Properties

**DOI:** 10.3390/ijms221910686

**Published:** 2021-10-01

**Authors:** Taku Shoji, Shunji Ito, Masafumi Yasunami

**Affiliations:** 1 Department of Material Science, Graduate School of Science and Technology, Shinshu University, Matsumoto 390-8621, Japan; 2 Graduate School of Science and Technology, Hirosaki University, Hirosaki 036-8561, Japan; itsnj@hirosaki-u.ac.jp; 3 Department of Chemical Biology and Applied Chemistry, College of Engineering, Nihon University, Koriyama 963-8642, Japan

**Keywords:** azulene, 2*H*-cyclohepta[*b*]furan-2-one, cycloaddition, enamine, silyl enol ether

## Abstract

A variety of synthetic methods have been developed for azulene derivatives due to their potential applications in pharmaceuticals and organic materials. Particularly, 2*H*-cyclohepta[*b*]furan-2-one and its derivatives have been frequently used as promising precursors for the synthesis of azulenes. In this review, we describe the development of the synthesis of azulenes by the reaction of 2*H*-cyclohepta[*b*]furan-2-ones with olefins, active methylenes, enamines, and silyl enol ethers as well as their reactivity and properties.

## 1. Introduction

**Scheme 1:**
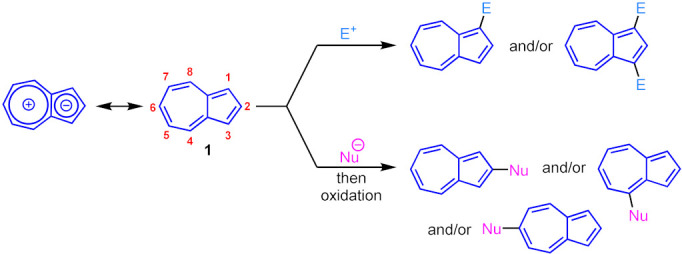
Resonance structure and general reactivity of azulene (**1**).

Azulene (**1**) is a 10 π-electron non-benzenoid aromatic hydrocarbon with a fused structure of five- and seven-membered rings, showing a deep blue coloration. The resonance structure of azulene contains ionic cyclopentadienide and tropylium substructures, resulting in electrophilic substitution reactions at the 1- and 3-positions [1] and nucleophilic addition reactions at the 2-, 4-, 6-, and 8-positions [2,3,4], along with the 2-position at the five-membered ring in some cases (Scheme 1).

**Figure 1:**
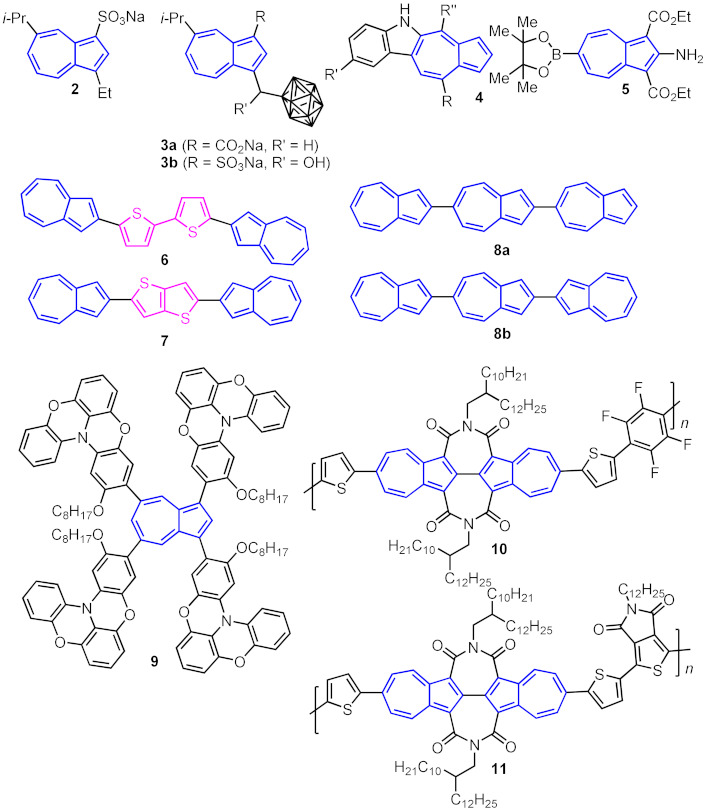
Azulene derivatives promising application to biologically active substances and organic materials.

Azulene derivatives are a promising class of compounds anticipated to have applications in pharmaceuticals and organic materials (Figure 1). In 1990, Yanagisawa, Yasunami, and their collaborators reported the preparation of various sodium sulfonates of alkylazulene derivatives and evaluated their pharmacological activities and clarified that the sulfonate of 1-ethyl-5-isopropylazulene **2** exhibited extremely high antiulcer activity [5]. Compound **2** is now frequently prescribed as a therapeutic agent for gastric ulcers. Nakamura and Yamamoto reported the azulene-substituted carborane derivatives **3a** and **3b** with high water solubility, which show high accumulation in B-16 melanoma cells *in vitro*, despite its low cytotoxicity, and was revealed as a promising boron carrier for neutron capture therapy [6]. The azuleno[6,5-*b*]indole derivatives **4** reported by Hong et al. have been evaluated for their antitumor activity and are revealed to exhibit good antitumor activity against a variety of cancer cells (melanoma, leukemia, lung, colon, kidney, ovary, brain, breast, and prostate) [7]. Lewis et al. reported the synthesis of AzuFluor® 483-Bpin **5** showing fluorescence upon the reaction with reactive oxygen species (ROS) and reactive nitrogen species (RNS) *in vivo*, which are associated with various diseases such as cancer and cardiovascular disease [8]. AzuFluor® 483-Bpin **5** is used to stain various living cells to show remarkable luminescence upon the reaction with intracellular ROS and RNS. Therefore, this azulene derivative **5** is expected to be applied for the direct detection of ROS and RNS in living cells.

Recently, azulene derivatives have also gained interest in the field of materials chemistry (Figure 1). Katagiri and co-workers have reported the preparation and characterization of 2-azulenyl group-substituted 2,2’-bithiophene **6**, thieno[3,2-*b*]thiophene **7**, [9] and terazulene derivatives **8a**,**b**, [10,11] which exhibit the properties of organic field-effect transistors (OFETs) with relatively high carrier mobility. Wakamiya and Scott et al. described the synthesis and properties of azulene derivative **9** possessing four oxygen-bridged triarylamines, and elucidated that this compound is an excellent hole transport material for perovskite solar cells (power conversion efficiency = 16.5%) [12]. Gao et al. investigated the thermal stability of conjugated polymers **10** and **11** composed of 2,2’-biazulene diimide units by thermogravimetric analysis (TGA) and found that these polymers are not decomposed until above 400 °C. Conjugated polymers **10** and **11** also represent excellent OFET performance with high electron mobility, and in particular, polymer **11** was found to be one of the best monopolar *n*-type polymers reported so far, functioning as a high-performance OFET [13]. 

**Scheme 2:**
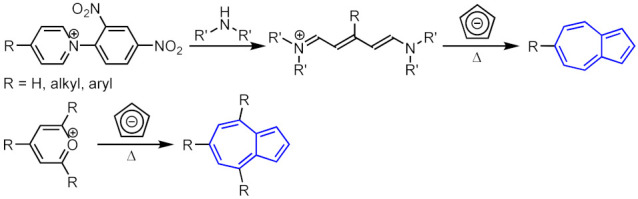
Synthesis of azulenes from pyridinium and pyrylium salts.

Although many methods for the synthesis of azulene derivatives have been reported, the methods described below are particularly popular because of their high yields and capability for large-scale synthesis. The effective and practical synthetic methods for azulene and its derivatives were developed in the 1950s independently by Ziegler and Hafner, and by Nozoe. The Ziegler–Hafner method, which involves the condensation of Zincke salts derived from either pyridinium [14,15] or pyrillium salts [16] with cyclopentadienide ions, allows the synthesis of parent azulene (**1**) and its alkyl and aryl derivatives on a large scale (Scheme 2). Ziegler–Hafner’s method is quite effective in the preparation of azulene derivatives with substituents at the seven-membered ring, and no better method except for the Ziegler–Hafner’s method has yet been reported for the synthesis of the parent azulene.

**Scheme 3:**
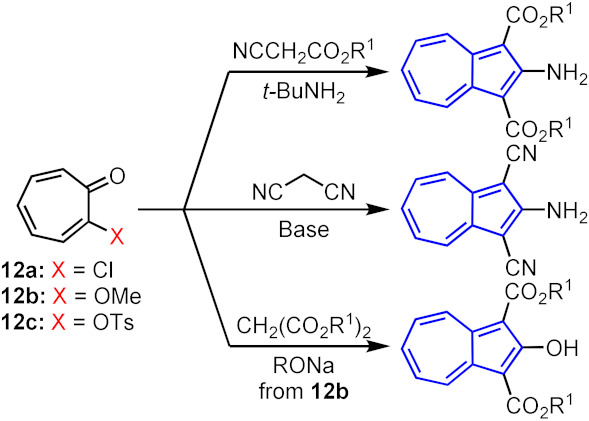
Synthesis of azulene derivatives from tropone derivatives **12**.

In the synthetic method of Nozoe and co-workers, the reaction of tropone derivatives **12** bearing a leaving group (e.g., halogen, methoxy, tosyloxy group) at the 2-position with active methylenes (e.g., cyanoacetate, malonate, and malononitrile) in the presence of a base provides 2-amino- and 2-hydroxyazulene derivatives in excellent yields (Scheme 3) [17]. This method is beneficial for the preparation of azulene derivatives having an amino or hydroxy group at the 2-position, which can be converted to the derivatives with other functional groups. In recent years, the synthesis of 2-aminoazulene derivatives with two butoxy esters has also been reported by the reaction of compound **12c** with butyl cyanoacetate in the presence of *tert*-butylamine (*t*-BuNH_2_) [18]. The conversion of the tropone derivatives to the azulene derivatives proceed via 2*H*-cyclohepta[*b*]furan-2-one and their analogous intermediates, as described in a later section. 

2*H*-Cyclohepta[*b*]furan-2-ones can also be used as useful starting materials for the synthesis of azulene derivatives with a variety of substituents and/or those with complex structures. As mentioned above, a variety of azulene derivatives have been prepared in recent years for the applications to pharmaceuticals and materials science, but for the applications preparation of more complex molecules is required. Although several reviews on the synthesis and reactivity of azulene and its derivatives have been reported, [19,20,21,22] there is still no comprehensive review that focuses on the synthesis using 2*H*-cyclohepta[*b*]furan-2-ones as starting materials. Therefore, we believe that a systematic review for the synthesis of azulenes using 2*H*-cyclohepta[*b*]furan-2-ones and their reactivity will be valuable for the future application of azulene derivatives to pharmaceuticals and organic materials. From these contexts, this review describes the progress in the synthesis of azulene derivatives starting from 2*H*-cyclohepta[*b*]furan-2-ones along with the reactivity and properties of the azulene derivatives prepared from 2*H*-cyclohepta[*b*]furan-2-ones.

## 2. Synthetic Method of 2*H*-cyclohepta[*b*]furan-2-ones

**Scheme 4:**
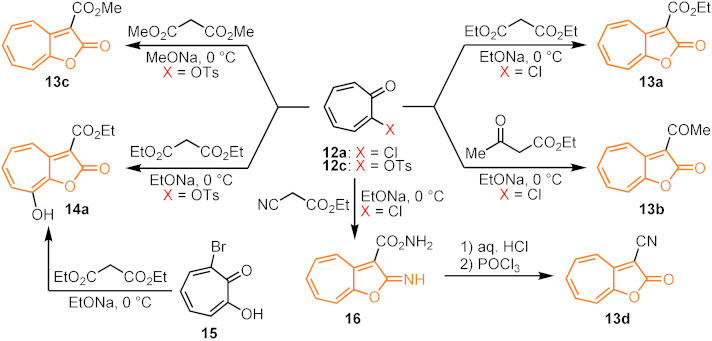
Preparation of 2*H*-cyclohepta[*b*]furan-2-ones with carbonyl and cyano functions at the 3-position.

Although there are several methods for the synthesis of 2*H*-cyclohepta[*b*]furan-2-ones, the most frequently adopted method is the reaction of tropone derivatives having a leaving group at the 2-position with active methylenes (Scheme 4). The reaction of 2-chlorotropone with diethyl malonate or ethyl acetoacetate in the presence of sodium ethoxide (EtONa) gives 3-ethoxycarbonyl and 3-acetyl derivatives **13a** and **13b** of 2*H*-cyclohepta[*b*]furan-2-one [23,24]. When tropolone tosylate (2-tosyloxytropone) **12c** is employed, the reaction with dimethyl malonate in the presence of sodium methoxide (MeONa) affords 3-methoxycarbonyl derivative **13c** as a major product [25]. In contrast, 8-hydroxy derivative **14a** is produced by the reaction of **12c** with diethyl malonate in the presence of EtONa. The difference in the reactivities is thought to be due to the slight difference in p*K*a of the solvents, i.e., methanol (p*K*a = 15.5) and ethanol (p*K*a = 16.0). 8-Hydroxy derivative **14a** is also produced by the reaction of 3-bromotropolone **15** with diethyl malonate in the presence of EtONa. Cyano derivative **13d** can be obtained by the reaction of **12a** with ethyl cyanoacetate in the presence of EtONa to give **16**, followed by hydrolysis and then dehydration of the amide group of **16** using phosphoryl chloride (POCl_3_).

**Scheme 5:**
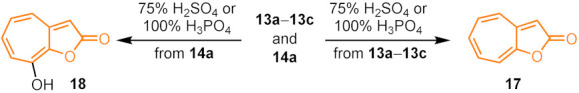
Decarboxylation and deacetylation of function at the 3-position.

**Scheme 6:**
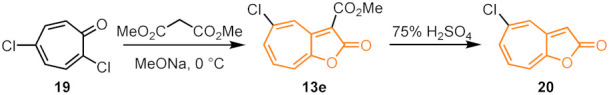
Preparation of 2*H*-cyclohepta[*b*]furan-2-one **13e** and following decarboxylation to give the derivative **20** with chlorine function at the 5-position.

When the carbonyl derivatives **13a**–**13c** and **14a** are heated in 75% sulfuric acid or 100% phosphoric acid (H_3_PO_4_), the decarboxylation and deacetylation proceed to provide their parent compounds **17** and **18** in good yields (Scheme 5).[26] The reaction of 2,5-dichlorotropone **19** derived from 5-chlorotropolone with dimethyl malonate yields 5-chloro-2*H*-cyclohepta[*b*]furan-2-one **13e**, which can also be converted to **20** via decarboxylation in a similar manner as described above (Scheme 6) [27].

**Scheme 7:**
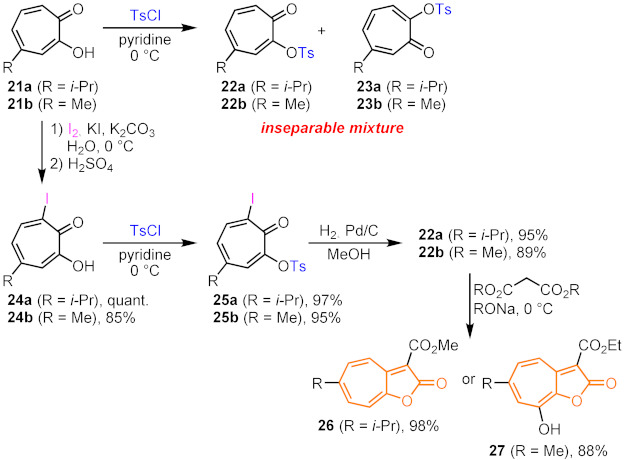
Selective synthesis of 2*H*-cyclohepta[*b*]furan-2-ones **26** and **27** with an alkyl function at the 6-position starting from hinokitiol **21a** and 4-methyltropolone **21b**.

Selective synthesis of 2*H*-cyclohepta[*b*]furan-2-ones **26** and **27** with an alkyl function at the 6-position starting from hinokitiol **21a** [28] or 4-methyltropolone **21b** [29] was established by a four-step procedure (Scheme 7). This is because direct tosylation of **21a** and **21b** produces an inseparable mixture of **22a**,**b** and **23a**,**b** due to inherent tautomerism, and besides, these are difficult to separate. However, this problem can be circumvented by the iodination of tropolones **21a** and **21b** prior to the tosylation. Iodination of **21a** and **21b** at the α-carbon converts to iodides **24a** and **24b**, which are then tosylated with *p*-toluenesulfonyl chloride to generate **25a** and **25b**, selectively, because of the steric hindrance of the substituted iodine. The iodine substituent of **25a** and **25b** is removed in high yield by catalytic hydrogenation to afford tosyloxytropones **22a** and **22b**, which react with malonate esters in the presence of sodium alkoxide yielding the corresponding 2*H*-cyclohepta[*b*]furan-2-ones **26** and **27** in excellent yields.

**Scheme 8:**
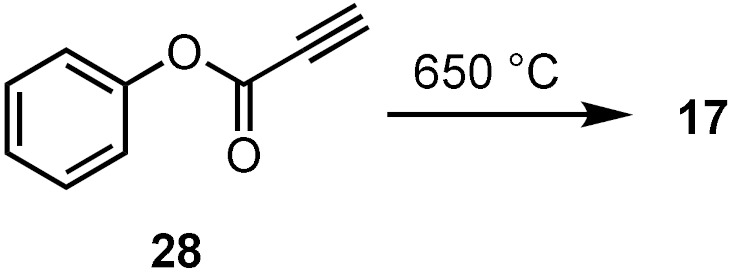
Preparation of 2*H*-cyclohepta[*b*]furan-2-one (**17**) from propynoic acid phenyl ester **28** by using FVP.

As one approach that does not use tropone derivatives, Trahanovsky et al. developed a method to convert phenol into 2*H*-cyclohepta[*b*]furan-2-ones, in which several propiolic acid phenyl esters are subjected to flash vacuum pyrolysis (FVP) at 650 °C yielding the corresponding products in 30–45% yields (Scheme 8) [30]. A similar method was developed by Hansen et al. for the preparation of polyalkylcyclohepta[*b*]furan-2-ones by the dynamic gas-phase thermo-isomerization (DGPTI) of polyalkylphenylpropiolates [31].

## 3. Synthesis of Azulenes by the Reaction of 2*H*-cyclohepta[*b*]furan-2-ones with Active Methylenes

The preparation of 2-amino- and 2-hydroxyazulenes by the reaction of 2*H*-cyclohepta[*b*]furan-2-ones with active methylenes has been reported, but there are not so many examples about the reports.

**Scheme 9:**
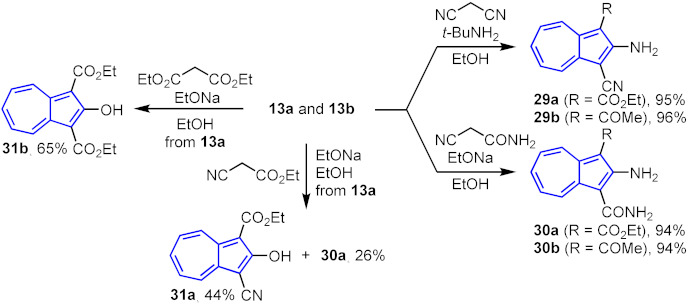
Reaction of 2*H*-cyclohepta[*b*]furan-2-ones **13a** and **13b** with active methylenes.

In 1971, Takase, Nozoe, and their collaborators reported the synthesis of 2-hydroxy- and 2-aminoazulene derivatives by the reaction of 2*H*-cyclohepta[*b*]furan-2-ones having a carbonyl group at the 3-position with active methylenes (Scheme 9) [23]. At that time, it was known that 2-methoxy- and 2-chlorotropones **12a** and **12b** react with active methylenes to give azulene derivatives directly, but the fact that the intermediates of this reaction are 2*H*-cyclohepta[*b*]furan-2-ones had not yet been clarified. Therefore, Takase et al. investigated the reaction of 2*H*-cyclohepta[*b*]furan-2-ones with active methylenes, such as malononitrile, cyanoacetamide, ethyl cyanoacetate, and diethyl malonate, to clarify the mechanism of azulene formation from the tropone derivatives.

The reaction of **13a** and **13b** with active methylenes takes place in the presence of EtONa or *t*-BuNH_2_ as a base to yield the 1,2,3-tri-substituted azulene derivatives. The outcome of the reaction between tropone derivatives and active methylenes proves that the intermediates for the formation of azulene derivatives are 2*H*-cyclohepta[*b*]furan-2-ones.

When **13a** and **13b** are reacted with malononitrile or cyanoacetamide, the corresponding 2-aminoazulene derivatives **29a**,**b** and **30a**,**b** are formed as the main products. The mixture of 2-amino- and 2-hydroxyazulenes **30a** and **31a** is formed by the reaction of **13a** with ethyl cyanoacetate, while the reaction with diethyl malonate furnishes 2-hydroxyazulene **31b** as the main product. The acetyl derivative **13b** is treated with ethyl cyanoacetate or diethyl malonate in the presence of EtONa giving a mixture including the 2-methylazulene derivatives.

**Scheme 10:**
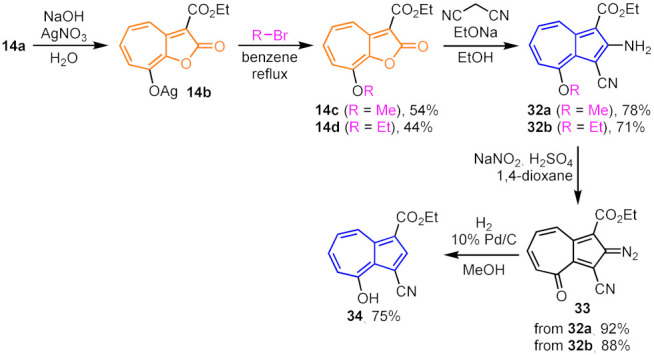
Diazotization of **32a** and **32b** prepared from **14a** to afford azulenequinone **33** converted to 4-hydroxyazulene **34**.

Nozoe et al. examined whether the products of the diazotization of 2-amino-3-cyano-4-alkoxyazulenes are azulenequinone or diazonium derivatives [32]. The precursor for the diazotization, 2-amino-3-cyano-4-alkoxyazulenes **32a** and **32b**, can be obtained in three steps from 8-hydroxy-2*H*-cyclohepta[*b*]furan-2-one **14a** (Scheme 10). After the conversion of **14a** to the silver salt **14b** by the treatment with silver nitrate in sodium hydroxide solution, the resulting **14b** is subjected to the reaction with MeBr or EtBr to afford the corresponding 8-methoxy and 8-ethoxy derivatives **14c** and **14d** in 54 and 44% yields, respectively. Similar to the method reported by Takase et al., condensation of **14c** and **14d** with malononitrile in the presence of EtONa provides the corresponding 2-amino-3-cyano-4-alkoxyazulenes **32a** and **32b** in good yields. Diazotization of **32a** and **32b** does not give diazonium salts, but affords azulenequinone **33** in excellent yield. Also, the regeneration of the azulene structure **34** from **33** is accomplished by catalytic hydrogenation in the presence of 10% Pd/C as a catalyst.

As an improvement on the method of Takase and co-workers, a procedure for the synthesis of 2-aminoazulene derivatives has been reported by the reaction of 2*H*-cyclohepta[*b*]furan-2-ones having various substituents at the 3-position with malononitrile under the milder basic conditions, i.e., in triethylamine [33]. Using this procedure, 2-aminoazulene derivatives **35a**–**35j** with various substituents at the 3-position can be obtained in excellent yields (85–93%, Table 1). Furthermore, this method requires a simple workup process because the products are obtained as pure precipitates and can be readily isolated by filtration. However, this reaction is successful only when the substituent at the 3-position is a relatively strong electron-withdrawing group, whereas 2*H*-cyclohepta[*b*]furan-2-ones substituted by iodine or methylsulfide results in quantitative recovery of the starting materials. When the substituent at the 3-position on the 2*H*-cyclohepta[*b*]furan-2-one is a formyl group, the reaction generates **36** in 92% yield, which is formed by the cooperation with the formation of azulene ring and Knoevenagel condensation [34].

## 4. Synthesis of Azulenes by the Reaction of 2*H*-cyclohepta[*b*]furan-2-ones with Electron-Rich Olefins and Their Analogues

2*H*-cyclohepta[*b*]furan-2-ones react with electron-rich olefins and their analogues, such as enol ethers, acetals, and fulvenes, to produce multiply functionalized azulenes.

**Scheme 11:**
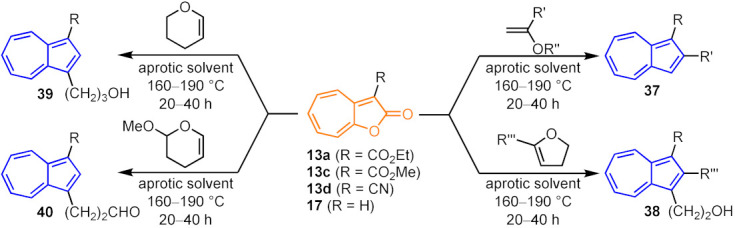
Synthesis of azulene derivatives **37**–**40** by the reaction of 2*H*-cyclohepta[*b*]furan-2-ones with enol ethers.

Nozoe and Wakabayashi et al. reported the synthesis of azulene derivatives with various functional groups by a [8 + 2] cycloaddition of 2*H*-cyclohepta[*b*]furan-2-ones with enol ethers (Scheme 11) [35]. Importantly, this method affords azulene derivatives in moderate to excellent yields, despite the need for high reaction temperatures, i.e., 160–190 °C, in aprotic solvents (tetrahydrofuran, acetonitrile, toluene, or in neat conditions). In the azulene synthesis by this reaction, the products are diversified depending on the enol ethers used. For instance, the reaction of 2*H*-cyclohepta[*b*]furan-2-ones with vinyl ethers affords 1,2-disubstituted azulenes **37**, whereas dihydrofurans react to yield the 1-azulenylethanol derivatives **38**. 1-Azulenylpropanols **39** are obtained by the reaction with dihydropyran, while the reaction with 2-methoxydihydropyran results in 1-azulenylpropanals **40**.

**Scheme 12:**
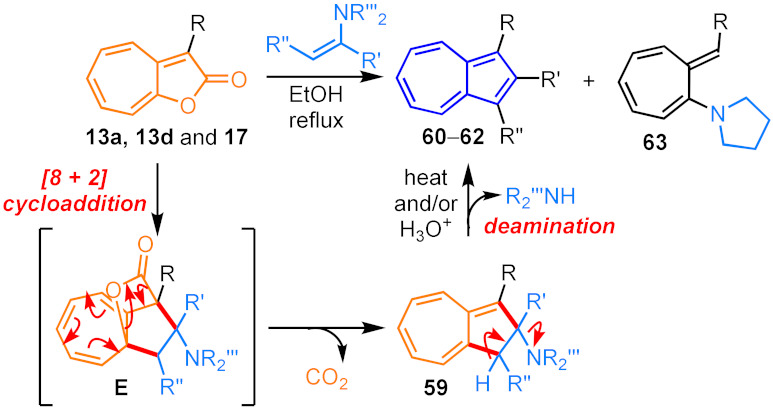
Synthesis of azulene derivatives **60**–**62** by the [8 + 2] cycloaddition of 2*H*-cyclohepta[*b*]furan-2-ones with enamines and its reaction mechanism.

**Scheme 13:**
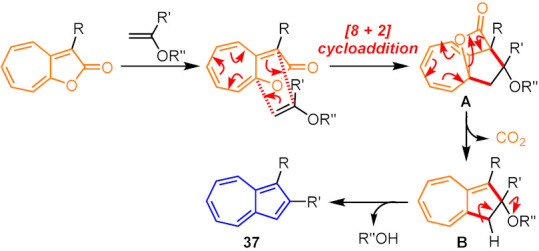
Reaction mechanism for the formation of azulene derivatives **37** by the reaction of 2*H*-cyclohepta[*b*]furan-2-ones with enol ethers.

The formation of azulene rings by the reaction of 2*H*-cyclohepta[*b*]furan-2-ones with enol ethers proceeds by [8 + 2] cycloaddition. The mechanism is similar to the reaction with enamines described below (see Scheme 12). The [8 + 2] cycloaddition of 2*H*-cyclohepta[*b*]furan-2-ones with enol ethers gives the strained intermediate **A**. Subsequently, **A** is decarboxylated to resolve the strain to form intermediate **B**, which is followed by the elimination of the alcohol to produce the azulene derivatives **37** (Scheme 13).

**Scheme 14:**
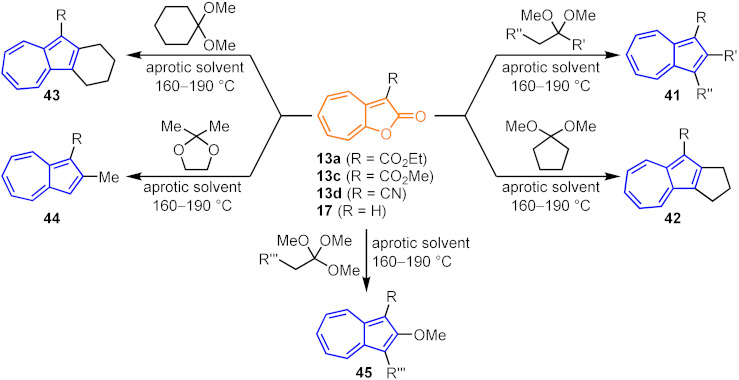
Synthesis of azulene derivatives **41**–**45** by the reaction of 2*H*-cyclohepta[*b*]furan-2-ones with acetals and orthoesters.

**Scheme 15:**
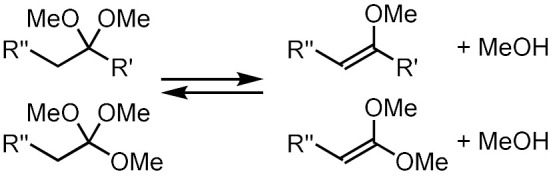
Equilibrium between acetals, orthoesters, and enol ethers under the high-temperature conditions.

Azulene derivatives with functional groups at the five-membered ring can also be synthesized by the reaction of 2*H*-cyclohepta[*b*]furan-2-ones with acetals prepared from aldehydes and ketones in neat or aprotic solvents under the heating conditions at 160–190 °C (Scheme 14). In this method, acetals prepared from cyclic ketones, such as cyclopentanone and cyclohexanone, are employed to obtain cycloalkane-fused azulenes **42** and **43**. This reaction is also applicable to the synthesis of 2-alkoxyazulenes **45** by using orthoesters as a reagent with low to excellent yields (11% to 99%) [36]. The formation of the azulene derivatives by this reaction can be explained by the same reaction mechanism as the reaction with enol ethers since acetals and orthoesters exist in equilibrium with enol ethers under the high-temperature conditions as shown in Scheme 15.

**Scheme 16:**
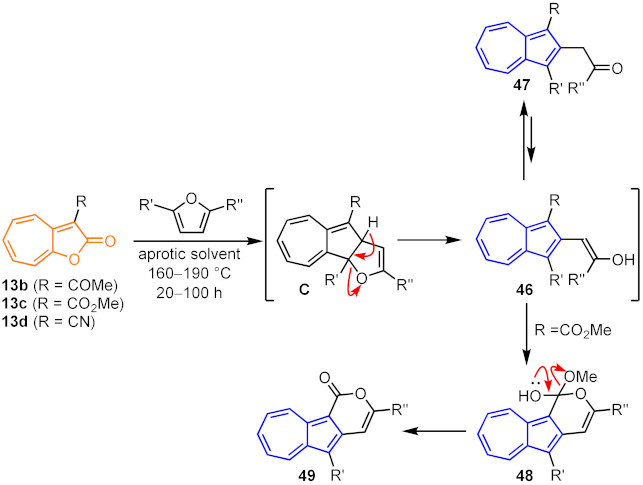
Synthesis of azulene derivatives **47** and **49** by the reaction of 2*H*-cyclohepta[*b*]furan-2-ones with furans.

Synthesis of azulene derivatives with a carbonyl substituent, such as acylmethyl or methoxycarbonyl methyl groups, at the 2-position has been achieved by the reaction of furan derivatives with 2*H*-cyclohepta[*b*]furan-2-ones (Scheme 16) [37]. In this synthesis, the yield of the products is affected by both the substituents on the 2*H*-cyclohepta[*b*]furan-2-ones and the furan derivatives, and the yield of the carbonyl derivatives **47** varies from 8 to 79% yields. Furthermore, when the substituent R on the 2*H*-cyclohepta[*b*]furan-2-ones is CO_2_Me, intramolecular cyclization of the presumed intermediate **46** occurs subsequently to afford the azulenes-fused δ-lactones **49** in 10–90% yields. The reaction mechanism for above is also shown in Scheme 16: furan reagent serves as an olefin and reacts with 2*H*-cyclohepta[*b*]furan-2-ones by [8 + 2] cycloaddition mode to furnish the adduct **C**, followed by the ring-opening of the adducted furan ring of **C** to produce the enol intermediate **46**, which tautomerizes eventually into carbonyl product **47**. In the case of 2*H*-cyclohepta[*b*]furan-2-ones with a methoxycarbonyl group at the 3-position, condensation of the ester function and the OH group of enol **46** takes place to give δ-lactones **49** by following the elimination of the methanol from the presumed addition intermediate **48**.

Yasunami, Takase, and co-workers reported the reaction of **13c** with 6,6-dimethylfulvene to give two types of cycloadducts, in which the products and their yields depend on the solvent employed (Table 2) [38]. In xylene, the reaction of **13c** with 6,6-dimethylfulvene gives the [8 + 2] cycloadduct, i.e., dihydroazulene derivative **50**, as a sole product in 35% yield (entry 1). On the other hand, the reaction in refluxing benzene gives **50** (21%) and the [4 + 2] cycloadduct **51** (28%) with almost the same production rate (entry 2). Furthermore, **51** is the major product in the reaction in ethanol at the reflux temperature (entry 3). When treated with 100% H_3_PO_4_ at 90 °C, the [8 + 2] cycloadduct **50** is converted to cyclopentadiene-fused azulene derivative **52** in 64% yield. These differences in the reactivities are also investigated in terms of theoretical calculations.

**Scheme 17:**
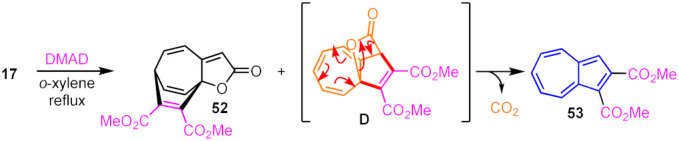
The reaction of 2*H*-cyclohepta[*b*]furan-2-one (**17**) with DMAD.

Electron-deficient olefins tend to cause the [4 + 2] cycloaddition at the seven-membered ring of 2*H*-cyclohepta[*b*]furan-2-one (**17**). Tomioka and Nitta investigated the reaction of **17** with dimethyl acetylenedicarboxylate (DMAD) to produce the [4 + 2] adduct **52** (71%) and azulene derivative **53** (9%) in a 7:1 ratio [39]. The reaction mechanism is discussed based on MNDO calculations, suggesting that **53** is formed via a [8 + 2] cycloaddition reaction of **17** to give intermediate **D**, followed by the decarboxylation (Scheme 17).

**Scheme 18:**
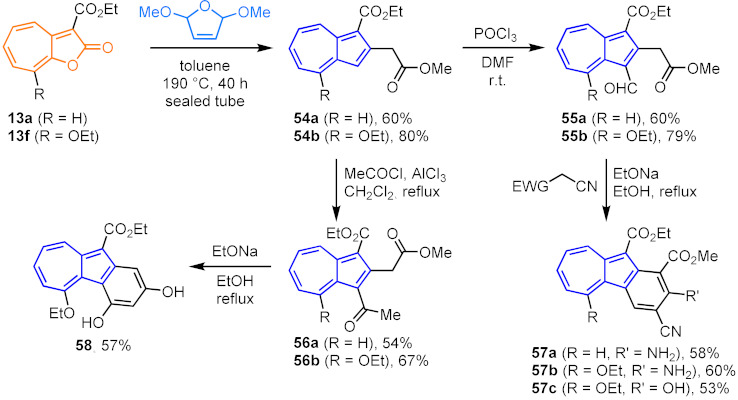
Synthesis of azulenes **54** and **55** with acylmethyl or methoxycarbonylmethyl functions and transformation to multiply functionalized benz[*a*]azulenes **57** and **58**.

Wu, Ku, and their collaborators reported the synthesis of azulene derivatives with acylmethyl or methoxycarbonylmethyl groups at the 2-position by mimicking the Nozoe’s method and their conversion to benz[*a*]azulene derivatives (Scheme 18) [40]. The reaction of **13a** and **13f** with 2,5-dimethoxy-2,5-dihydrofuran under the sealed tube conditions provides **54a** and its 4-ethoxy derivative **54b** in 60 and 80% yields, respectively. These derivatives can be carbonylated at the 1-position of the azulene ring with good yields by Vilsmeier formylation or Friedel-Crafts acylation reactions to give **55a**,**b** and **56a**,**b**. The formyl derivatives **55a** and **55b** react with active methylenes in the presence of EtONa yielding multiply functionalized benz[*a*]azulenes **57** in moderate to good yields. Whereas *m*-cresol-fused benz[*a*]azulene **58** can be obtained in 65% yield by EtONa-mediated intramolecular cyclization of acyl derivative **56b**.

## 5. Synthesis of Azulenes by the Reaction of 2*H*-cyclohepta[*b*]furan-2-ones with Enamines

Currently, the most frequently used procedure for azulene synthesis using 2*H*-cyclohepta[*b*]furan-2-ones as starting materials is the Yasunami-Takase’s method by the reaction with enamines. In the 1970s and 1980s, they reported the efficient synthesis of azulene derivatives by the reaction of 2*H*-cyclohepta[*b*]furan-2-ones with enamines prepared from various aldehydes or ketones.[41]. In this reaction, the [8 + 2] cycloaddition of 2*H*-cyclohepta[*b*]furan-2-ones with enamines affords initially the strained intermediate **E**, and subsequent decarboxylation from the intermediate **E** yields the aminohydroazulene intermediate **59** (Scheme 12). The aminohydroazulene **59** can be isolated as a stable compound in some cases (see below). Finally, the reaction is completed by the aromatization of **59** by the deamination to give the thermodynamically stable azulenes **60**–**62**. This synthetic method is one of the effective ways to introduce various substituents to the five-membered ring during the formation of an azulene ring.

**Figure 2:**
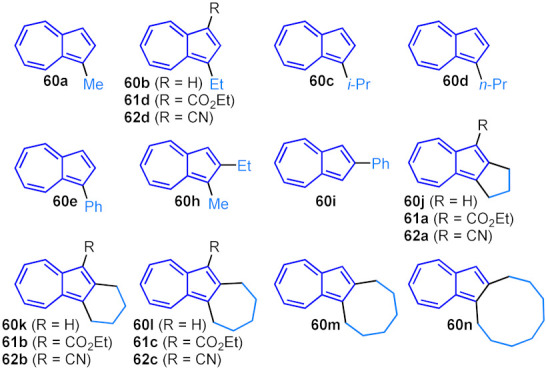
Structure of azulenes obtained by the [8 + 2] cycloaddition of 2*H*-cyclohepta[*b*]furan-2-ones with enamines.

In the synthesis of azulenes by the [8 + 2] cycloaddition of 2*H*-cyclohepta[*b*]furan-2-ones with enamines, the yield and reactivity depend on both the substituent R on 2*H*-cyclohepta[*b*]furan-2-ones, amines, and the carbonyl compounds used in the preparation of the enamines (Table 3, Figure 2). In general, enamines prepared from aldehydes are more reactive toward 2*H*-cyclohepta[*b*]furan-2-ones than those prepared from ketones. Furthermore, the reaction rate of pyrrolidine-substituted enamines with 2*H*-cyclohepta[*b*]furan-2-ones is much faster than that of morpholine enamines [42,43]. The reaction with the enamines derived from cyclic ketones gives azulene derivatives, in which the cycloalkanes are fused to the five-membered ring. However, the reaction of 2*H*-cyclohepta[*b*]furan-2-ones with the enamines prepared from cyclopentanones frequently yields aminohydroazulenes **59** as the main products, but **59** can be readily transformed into azulene derivatives by heating under the acidic conditions (Scheme 12). When pyrrolidine enamines are reacted with 2*H*-cyclohepta[*b*]furan-2-ones possessing an electron-withdrawing substituent as R, the yield of azulene derivatives **60**–**62** is reduced, as the result on the reaction of 2*H*-cyclohepta[*b*]furan-2-ones with pyrrolidine eliminated from the enamine to give 1-pyrrolidinylheptafulvenes **63** and insoluble resinous products [44]. In contrast, the reaction with morpholine enamines does not cause such undesired reactions and often results in good yields of 60–62%. Enamines, which are prepared from phenylacetaldehyde and acetophenone, conjugated with an aryl group are resistant to the reaction with 2*H*-cyclohepta[*b*]furan-2-ones and tend to require longer reaction times. However, the silyl enol ether prepared from acetophenone readily reacts with 2*H*-cyclohepta[*b*]furan-2-ones, giving the corresponding 2-phenylazulenes in excellent yield, despite requiring a high reaction temperature (see Section 7).

**Scheme 19:**
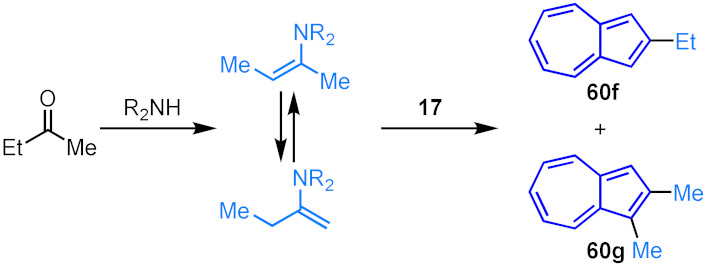
The reaction of 2*H*-cyclohepta[*b*]furan-2-one (**17**) with the enamines prepared from 2-butanone.

Enamines prepared from aldehydes produce 1-alkylazulenes, while enamines derived from ketones provide 1,2-dialkylazulenes and 2-alkylazulenes or a mixture thereof. When the enamines prepared from asymmetric dialkyl ketones such as 2-butanone are conducted, the reaction with **17** yields a mixture of **60f** and **60g** because of the existence of the tautomers of enamines (Scheme 19; Table 3, entries 6 and 16).

**Scheme 20:**
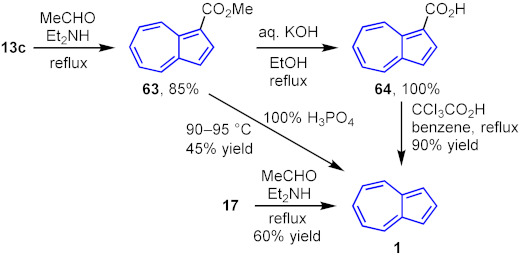
Synthesis of parent azulene (**1**) from 2*H*-cyclohepta[*b*]furan-2-ones **13c** and **17**.

The reaction of 2*H*-cyclohepta[*b*]furan-2-ones with enamines can be applied to the synthesis of parent azulene (**1**) (Scheme 20) [45]. The reaction of **17** with acetaldehyde in the presence of a solvent amount of diethylamine affords parent azulene (**1**) in 60% yield. However, when **13c** is treated under similar conditions, **63** with methoxycarbonyl substituent is obtained in 85% yield, which hydrolyzes with aqueous potassium hydroxide (KOH), leading to the carboxylic acid **64** in quantitative yield (100%). Eventually, **64** is transformed to **1** in 90% yield by the decarboxylation by the treatment with trichloroacetic acid (CCl_3_CO_2_H). The three-step synthesis of parent azulene (**1**) is more efficient than the direct preparation from **17**, since the overall yield is much higher (three-steps, 77% yield).

The [8 + 2] cycloaddition reaction of 2*H*-cyclohepta[*b*]furan-2-ones with enamines can be developed for the construction of azulene derivatives with extended conjugation π-electron systems. Therefore, a variety of π-expanded azulene derivatives have been prepared by using such reactions and their properties are elucidated.

**Scheme 21:**
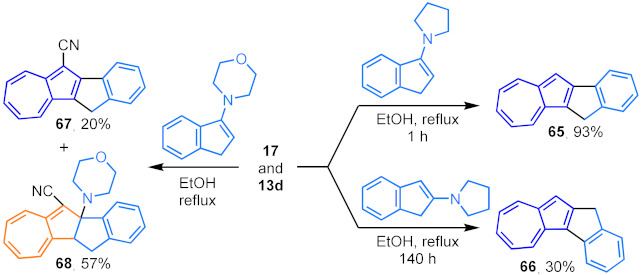
Synthesis of indenoazulenes **65**–**67** by the reaction of 2*H*-cyclohepta[*b*]furan-2-ones **17** and **13d** with enamines prepared from indanones.

Indenoazulenes **65**–**67** can be obtained by the reaction of 2*H*-cyclohepta[*b*]furan-2-ones **17** and **13d** with the enamines prepared from the corresponding indanones; however, the reaction time and product yield are highly dependent on the structure of the enamines employed (Scheme 21) [46]. The enamine prepared from 1-indanone and pyrrolidine reacts readily (within 1 hour) with **17** in ethanol under the reflux condition to provide indeno[2,1-*a*]azulene (**65**) in 93% yield. In contrast, the formation of indeno[1,2-*a*]azulene (**66**) by the reaction of **17** with the enamine prepared from 2-indanone is very slow (140 hours) and the yield is rather low (30%). Under the similar reaction conditions, **13d** reacts with the enamine prepared from 1-indene and morpholine affording 5-cyanoindeno[2,1-*a*]azulene (**67**) and dihydroazulene derivative **68** in 20 and 57% yields, respectively.

**Scheme 22:**
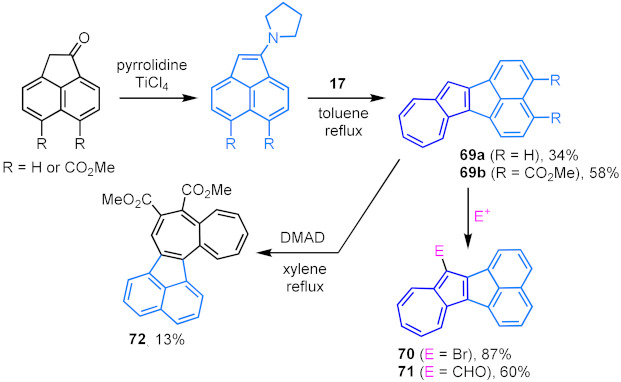
Synthesis of azuleno[1,2-*a*]acenaphthylenes **69**–**71** and heptalene derivative **72**.

Kuroda and Yasunami et al. have synthesized azuleno[1,2-*a*]acenaphthylenes **69**–**71** from **17** and discussed their aromaticity in terms of the bond-length alternation observed in ^1^H NMR spectra, as well as their reactivity (Scheme 22) [47]. The [8 + 2] cycloaddition reaction of **17** with the enamine prepared from acenaphthen-1-one and pyrrolidine leads to azuleno[1,2-*a*]acenaphthylene **69a** in 34% yield. A similar procedure can be extended to dimethoxycarbonyl derivative of acenaphthen-1-one to furnish **69b** in 58% yield. The ^1^H NMR chemical shifts of these compounds at the azulene moiety are almost identical to those of the parent azulene, and no significant bond-length alternation is observed from their ^1^H NMR spectra. Therefore, these results conclude that **69a** and **69b** are molecules composed of two independent 10 π-electrons, i.e., azulene and naphthalene, rather than acting as an estimated 20 π-electron system. Reactions of **69a** with electrophiles are also investigated; the reaction with bromine (Br_2_) gives **70** in 87% yield, and formyl derivative **71** is obtained in 60% yield by the reaction with orthoformate in the presence of BF_3_∙OEt.

In the reaction of **69a** with DMAD, the [2 + 2] cycloaddition proceeds initially to form the four-membered ring intermediate, followed by the retroelectrocyclization reaction yielding acenaphthyleno[1,2-*d*]heptalene **72** in 13% yield. Contrary to **69a** and **69b**, the ^1^H NMR spectrum of heptalene **72** shows a pronounced bond-length alternation in attributed to its non-aromatic nature.

**Scheme 23:**
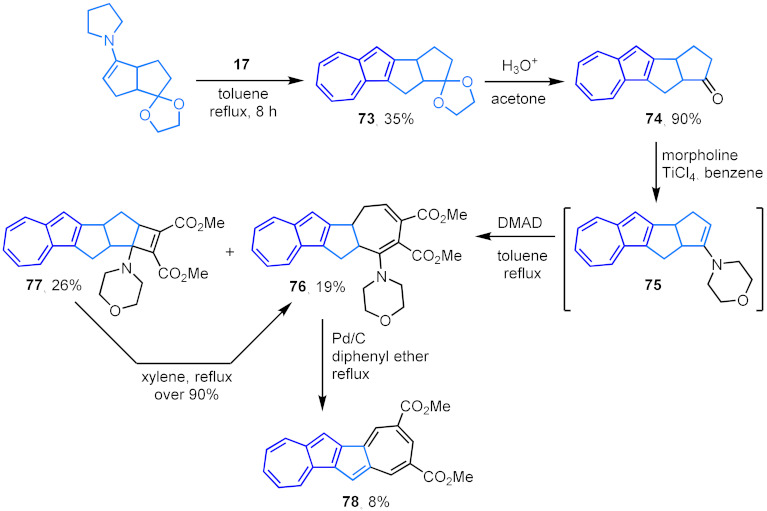
Synthesis of dimethyl azuleno[1,2-*b*]azulene-2,4-dicarboxylate **73**.

Synthesis of azuleno[1,2-*b*]azulene derivatives starting from **17** was established by Kuroda and Yasunami et al. in 1986 (Scheme 23) [48]. The reaction of **17** with the enamine in toluene at the reflux temperature yields **73** in 35% yield. Hydrolysis of the acetal moiety of **73** with HCl in acetone leads to ketone **74** in over 90% yield, and the reaction of **74** with morpholine in the presence of titanium tetrachloride (TiCl_4_) affords enamine **75**, which is unstable to the moisture. Enamine **75** is reacted with DMAD in a [2 + 2] cycloaddition to provide cycloadduct **77** in 26% yield, along with **76** in 19% yield. The cycloadduct **77** is transformed into **76** in refluxing xylene in more than 90% yield. Eventually, dehydrogenative aromatization of **76** with palladium-carbon in diphenyl ether under the reflux condition results in azuleno[1,2-*b*]azulene-2,4-dicarboxylate **78**, accompanying the rearrangement of an ester group, in 8% yield. The UV-visible absorption (UV/Vis) spectrum of **78** exhibits an absorption maximum in the near-infrared region at around λ_max_ = 1200 nm, suggesting that the conjugated system is largely extended.

**Scheme 24:**
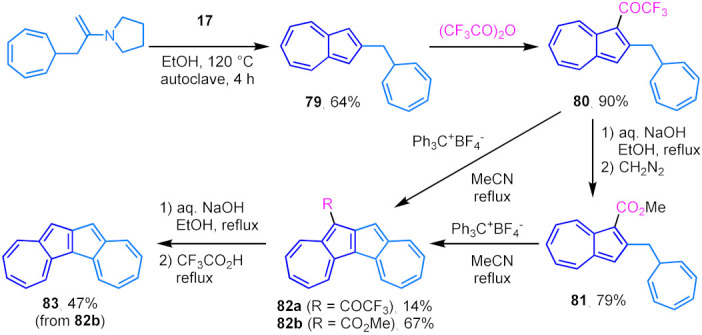
Synthesis of azuleno[1,2-*a*]azulenes **82a**,**b** and **83**.

Nitta et al. reported the preparation of azuleno[1,2-*a*]azulenes **82a**,**b** and **83** via [8 + 2] cycloaddition of **17** with an enamine (Scheme 24) [49]. The enamine was prepared by the condensation of 7-(2-oxo-propyl)-1,3,5-cycloheptatriene with pyrrolidine in the presence of the catalytic amount of *p*-toluenesulfonic acid and molecular sieves, which is subjected to the cycloaddition with **17** under the autoclave condition to give **79** in 64% yield. The azulene **79** reacts with trifluoroacetic anhydride [(CF_3_CO)_2_O] at 0 °C to give **80** in 90% yield. Treatment of **80** with NaOH in refluxing ethanol results in hydrolysis to afford the carboxylic acid, which is subsequently converted to the ester **81** (two-steps, 79% yield) by the treatment with diazomethane (CH_2_N_2_). Oxidative intramolecular cyclization of **80** and **81** gives the corresponding azuleno[1,2-*a*]azulenes **82a** and **82b**: treatment of **80** with 4 equivalents of triphenylcarbenium tetrafluoroborate (Ph_3_C^+^BF_4_^−^) in refluxing acetonitrile gives **82a** with a trifluoroacetyl group in 14% yield. Similarly, the reaction with **81** provides the methoxycarbonyl derivative **82b** in 67% yield. The methoxycarbonyl derivative **82b** was hydrolyzed to carboxylic acid using sodium hydroxide, and the subsequent decarboxylation in CF_3_CO_2_H furnished the parent derivative **83** in 47% yield.

The spectroscopic properties of these azuleno[1,2-*a*]azulenes **82a**,**b** and **83** have been characterized in terms of ^1^H NMR and UV/Vis spectra, and theoretical calculations. These results clearly show that azuleno[1,2-*a*]azulenes behave not as a 18 π aromatic system, but as a derivative of two independent fused azulene rings.

**Scheme 25:**
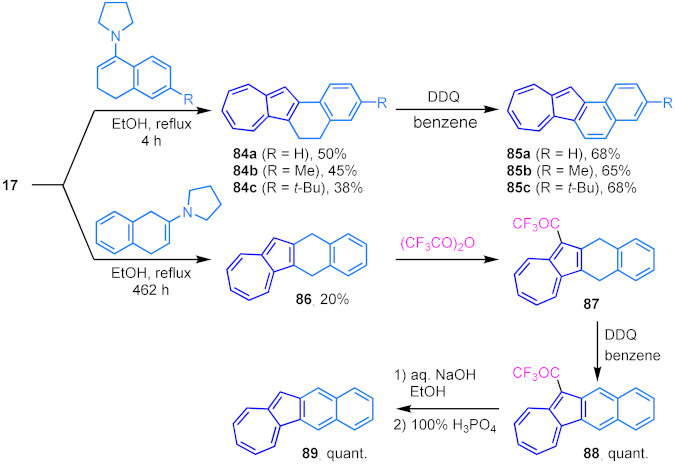
Synthesis of naphth[2,1-*a*]- and naphth[2,3-*a*]azulenes **85**, **88**, and **89**.

Yasunami et al. successfully synthesized naphth[2,1-*a*]- and naphth[2,3-*a*]azulenes **85**, **88**, and **89** via the corresponding dihydronaphthoazulenes from **17** as the starting material (Scheme 25) [50]. 2*H*-Cyclohepta[*b*]furan-2-one (**17**) reacts with the enamine prepared from 1-tetralone and pyrrolidine in refluxing ethanol to afford 5,6-dihydronaphth[2,1-*a*]azulene **84a** in 50% yield. Under the similar reaction conditions, the reaction of **17** with the enamines prepared from 7-methyl and 7-*tert*-butyl derivatives of 1-tetralone furnishes the corresponding 2-methyl and 2-*tert-*butyl derivatives **84b** and **84c** in 45 and 38% yields, respectively. When **84a**, **84b**, and **84c** are treated with 2,3-dichloro-5,6-dicyano-*p*-benzoquinone (DDQ), the dehydrogenative aromatization reaction proceeded to provide the corresponding naphth[2,1-*a*]azulenes **85a**, **85b**, and **85c** in 68, 65, and 68% yields, respectively. The compound **85a** has also been prepared recently by Murai, Takai, and co-workers by the intramolecular cyclization of 2-phenylazulene derivatives [51].

The reaction of the enamine prepared from 1-tetralone with **17** is completed within four hours, while the reaction with the enamine prepared from 2-tetralone requires a longer time (462 hours) and the yield of dihydronaphthoazulene **86** is also low (20% yield). Dehydrogenative aromatization of **86** with DDQ is difficult, so naphth[2,3-*a*]azulene **89** should be synthesized in a stepwise manner. The trifluoroacetyl derivative **87** obtained by the reaction of **86** with (CF_3_CO)_2_O is aromatized by DDQ to afford **88** in quantitative yield. The carboxylic acid obtained by the hydrolysis of **88** with sodium hydroxide in ethanol is decarboxylated with 100% H_3_PO_4_ to produce **89**, almost quantitatively.

**Scheme 26:**
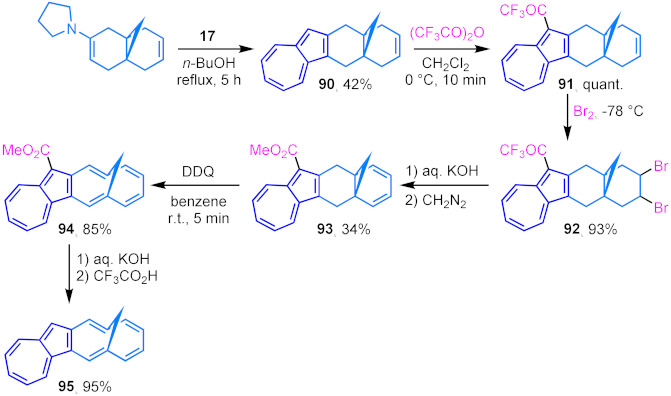
Synthesis of 2,7-methanocyclodec[*a*]azulenes **94** and **95**.

1,6-Methano[10]annulene is a class of molecules that satisfy Hückel’s rule. Various fused derivatives by aromatic and heterocyclic rings have been prepared and examined from the viewpoint of their characteristic aromaticity [52,53,54]. In 1994, Nitta et al. reported the synthesis and properties of 1,6-methano[10]annulenes fused to an azulene ring, namely, 2,7-methanocyclodec[*a*]azulenes **94** and **95**, which are prepared in a five- or six-step procedure from **17** as the starting material (Scheme 26) [55]. The [8 + 2] cycloaddition of enamine with **17** gives **90** in 42% yield, which is then converted to **91**, quantitatively, by the treatment with (CF_3_CO)_2_O to protect the 1-position of the azulene ring. Bromination of **91** at −78 °C affords **92** in 93% yield, subsequent treatment of **92** with an aqueous KOH takes place with the hydrolysis of the trifluoroacetyl group and the E2-type debromination, simultaneously, to produce a carboxylic acid derivative. The esterification of the carboxylic acid by CH_2_N_2_ gives **93** in 34% yield. Oxidation of **93** with DDQ in benzene furnishes the dehydrogenated product **94** immediately (within 5 min) in 85% yield. Decarboxylation product **95** is obtained by the hydrolysis of **94** to a carboxylic acid using aqueous KOH, followed by the trifluoroacetic acid-mediated decarboxylation in 95% yield.

In the ^1^H NMR spectra, the chemical shifts of the bridging methylenes of **94** and **95** are largely shielded by the ring-current and are observed at δ = −0.07 ppm and −0.34 ppm, respectively. This indicates that the methano[10]annulene moiety in these compounds possesses sufficient aromatic character. Furthermore, even though the vicinal coupling constants of the methano[10]annulene moiety of **94** and **95** closely resemble each other, those of the azulene moiety suggest the contribution of a distinct bond-length alternation. These results confirm the quite small contribution of the 18 π electron system in **94** and **95**.

**Scheme 27:**
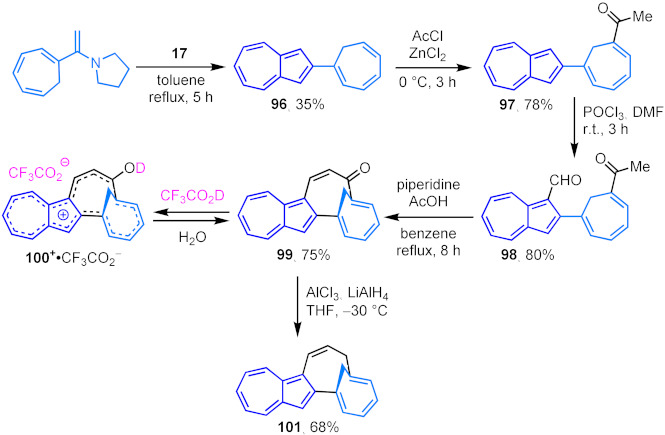
Synthesis of azulenoannulenes **99** and **101**, and their precursors.

In 1989, there were no reports for the 18 π-electron compounds with bridged annulene architectures. To construct such a molecule, Kuroda and co-workers investigated the synthesis of azulenoannulenes **99** and **101** and clarified their electronic properties (Scheme 27) [56].

The reaction of **17** with the enamine prepared from 1-acetylcyclohepta-1,3,5-triene and pyrrolidine in refluxing toluene affords 2-(cyclohepta-1,3,5-trienyl)azulene **96** in 35% yield. Acetylation of **96** is achieved by the treatment with acetyl chloride in the presence of zinc chloride in dichloromethane to give **97** in 78% yield. Compound **97** is subjected to Vilsmeier reaction affording **98**, which is further converted by intramolecular aldol condensation to the bridged-ring-fused azulene derivative **99**.

In CDCl_3_, the protons of the bridging methylene of **99** appear at δ = 3.58 and 1.03 ppm, which resonate at the lower fields than those of 4,9-methano[11]annulenone. On the other hand, the chemical shifts of the protons of the bridging methylene of the azulenylium ionic species **100^+^** produced in CF_3_CO_2_D are observed at the higher fields (δ = 1.34 and 0.38 ppm) than those of **99**. These results imply that the cationic species **100^+^** serve as bridged annulene derivatives of the 18 π-electron system in the acidic medium, even though the contribution of the 18 π-electron system is small in the neutral media. Reduction of **99** with LiAlH_4_ in THF in the presence of AlCl_3_ yields **101** in 68% yield, but the cationic species derived from **101** has not been obtained so far.

**Scheme 28:**
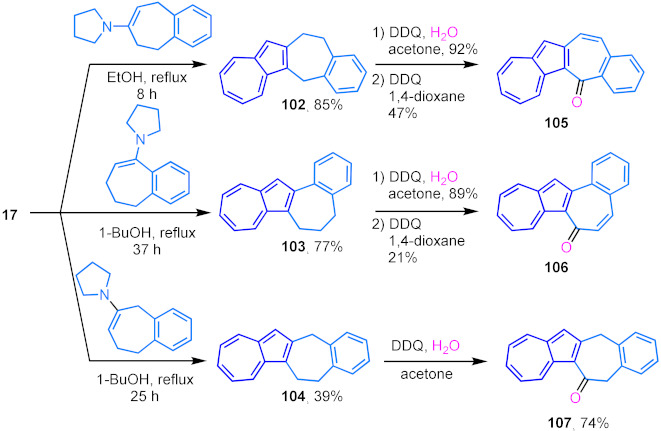
Synthesis of azulenobenzotropones **105** and **106**, and their related compounds.

In 2002, Nitta and co-workers prepared a series of azulenobenzotropones **105** and **106** to assess their reactivity and properties [57]. The [8 + 2] cycloaddition reaction of **17** with the enamines prepared from benzocycloheptanones gives the corresponding benzocycloheptazulenes **102**–**104** (Scheme 28). In these reactions, **102** (85%) and **103** (77%) are obtained in good yields, but the yield of **104** was rather low (39%). The reason for this is explained by the theoretical calculations of the enamines used. To introduce a carbonyl group to the fused-cycloheptane moiety, **102**–**104** are treated with DDQ in aqueous acetone to afford the corresponding carbonyl compounds in good to excellent yields (74−92%). Further oxidation of the carbonyl compounds by DDQ in refluxing 1,4-dioxane induces the aromatization to give the corresponding azulenobenzotropones **105** (47%) and **106** (21%) in moderate yields. As described later, these derivatives have also been converted into benzocyclohept[*a*]azulenylium ions and their aromaticity are evaluated from the viewpoint of the ^1^H NMR spectra. The ^1^H NMR spectra of **105** and **106** show the lower magnetic field shift in most of the proton signals in CF_3_CO_2_D, compared to those in CDCl_3_, attributed to the protonation of the carbonyl oxygen and the proton-deuterium exchange at the five-membered ring of the azulene moiety.

**Scheme 29:**
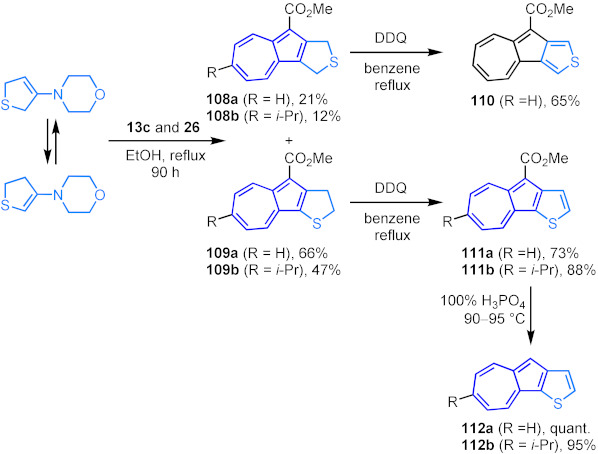
Synthesis of azuleno[1,2-*c*]- and azuleno[1,2-*b*]thiophenes **110**, **111a**,**b**, and **112a**,**b**.

Azulene derivatives fused with a heterocycle can also be prepared from 2*H*-cyclohepta[*b*]furan-2-ones. In 1983, Fujimori, Yasunami, and co-workers reported the synthesis of azuleno[1,2-*b*]- and azuleno[1,2-*c*]thiophenes **110**, **111a**,**b**, and **112a**,**b** starting from the reaction of 2*H*-cyclohepta[*b*]furan-2-ones with a mixture of enamines prepared from 3-oxotetrahydrothiophene (Scheme 29) [58,59]. The reactions of **13c** and **26** with the enamines prepared from 3-oxotetrahydrothiophene and morpholine in ethanol under the reflux condition for 90 hours gives dihydroazuleno[1,2-*c*]- and dihydroazuleno[1,2-*b*]thiophenes **108a**,**b** and **109a**,**b**, respectively. In this reaction, employing the enamines prepared with pyrrolidine leads to unsuccessful results. Dehydrogenation of dihydroazulenothiophenes **108a**,**b** and **109a**,**b** by the treatment with DDQ in refluxing benzene, followed by the removal of the precipitated hydroquinone, provides the corresponding azulenothiophenes **110** and **111a**,**b**. When **111a** and **111b** are heated in 100% H_3_PO_4_ at 90–95 °C, a decarboxylation reaction occurs to produce azuleno[1,2-*b*]thiophenes **112a** and **112b** in almost quantitative yields. These azuleno[1,2-*b*]thiophenes **111a**,**b** and **112a**,**b** are very stable at room temperature, while azuleno[1,2-*c*]thiophene **110** is extremely unstable under the ambient condition.

**Scheme 30:**
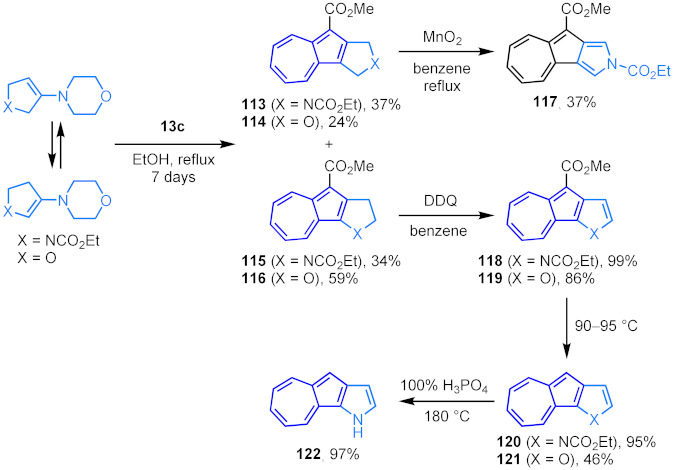
Synthesis of azuleno[1,2-*b*]pyrroles and furans **117**–**122**.

A similar pathway to the synthesis of azulenothiophenes has been adapted for the preparation of azulenopyrroles and furans **117**–**122** (Scheme 30) [60]. The reaction of the enamines, which are obtained by condensation of *N*-ethoxycarbonyl-3-oxopyrrolidine or 3-oxotetrahydrofuran with morpholine, with **13c** gives dihydroazulenopyrroles or furans **113**–**116** after seven days in refluxing ethanol. Azuleno[1,2-*b*]pyrrole and furan **118** and **119** can be obtained in 99 and 86% yields, respectively, by the aromatization of the corresponding dihydro derivatives **115** and **116** with DDQ. When **113** is treated with manganese dioxide in benzene, azuleno[1,2-*c*]pyrrole **117** is formed in 37% yield, while the corresponding furan derivative **114** does not show the aromatization under the similar reaction conditions. When **118** is treated in 100% H_3_PO_4_ at 90 °C, decarboxylation occurs only on the azulene ring to afford **120** in 95% yield, whereas by further increasing the reaction temperature (180 °C), the decarboxylation of the ester group on the nitrogen takes place to provide azuleno[1,2-*b*]pyrrole **122** in 97% yield. When a similar reaction is applied to **119** at 90–95 °C, azuleno[1,2-*b*]furan **121** is obtained in 46% yield. Azuleno[1,2-*b*]furan and pyrrole **121** and **122** suggested a slight decrease in the aromaticity of these compounds compared to that of the parent azulene, since the coupling constants of these derivatives in ^1^H NMR spectra show a distinct bond-length alternation at the seven-membered ring.

## 6. Reactivity and Properties of Azulene Derivatives Prepared from 2*H*-cyclohepta[*b*]furan-2-ones

The cycloalkane-fused azulenes produced by the reaction of 2*H*-cyclohepta[*b*]furan-2-ones with the enamines prepared from cyclic ketones can be derivatized by _O_xidation, condensation, aromatization reactions, and so on.

**Scheme 31:**
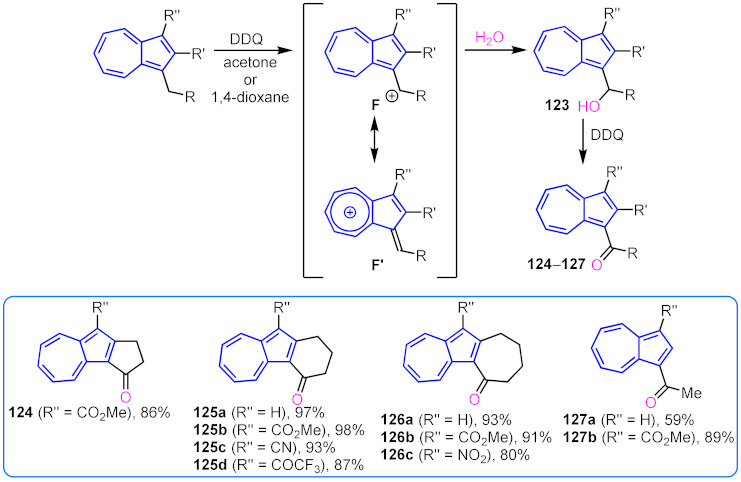
Oxidation of the α-methylene at the 1-position of the azulene ring with DDQ.

**Scheme 32:**
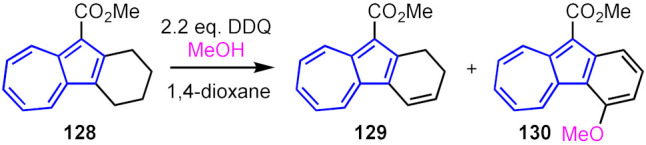
Oxidation of the α-methylene of **128** with DDQ in the presence of MeOH.

The oxidation of alkyl groups on the azulene ring to a carbonyl group has been very difficult because azulene derivatives have less tolerance to commonly used oxidizing reagents, such as chromic acid, nitric acid, and permanganic acid. However, Yasunami et al. developed a facile method to transform the α-methylene group of an alkyl group on an azulene ring into a carbonyl group by the treatment with DDQ [61]. The treatment of the cycloalkane-fused azulenes and 1-alkylazulenes with 2.2 equivalents of DDQ in acetone containing 10% water provides the corresponding azulenes **124**–**126** fused to a cyclic ketone and **127** in high yields (Scheme 31). In the case of 1-alkylazulenes with an electron-withdrawing trifluoroacetyl or nitro group at the 3-position, the oxidation of the α-methylene group at the 1-position of the azulene ring is rather slow. Furthermore, the alkyl group at the 2-position of the azulene ring is not oxidized by the reaction with DDQ in a similar manner. When 1-alkylazulenes are treated with 1.2 equivalents of DDQ in aqueous acetone give alcohols **123** in low yield, which treated with DDQ furnishes the carbonyl derivative in quantitative yield. These findings indicate that alcohols **123** should be the intermediate in the oxidation of the α-methylene group with DDQ. The treatment of **128** with DDQ in 1,4-dioxane containing methanol results in the generation of **129** and **130** (Scheme 32), but the yields have not appeared in the literature.

From the above results, the reaction mechanism can be drawn as follows: the hydride ion is abstracted from the α-methylene at the 1-position of the azulene ring by DDQ to form a cationic intermediate **F**, which is stabilized by the resonance structure where the seven-membered ring of the azulene moiety forms a tropylium ion substructure **F’**. In acetone or 1,4-dioxane, the nucleophilic addition of water or methanol to the generated cations forms the corresponding intermediates **123**, which are further oxidized by DDQ to form 1-carbonylazulenes **124**–**127**.

**Scheme 33:**
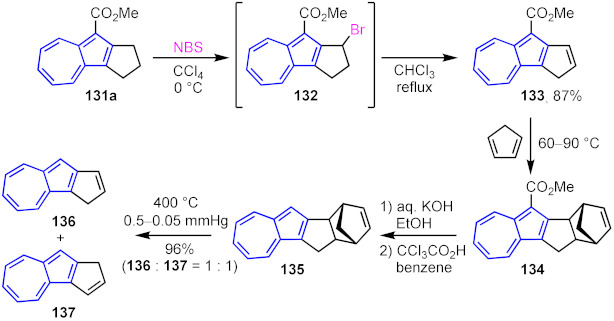
Synthesis of cyclopent[*a*]azulenes **136** and **137**.

Azulene-fused aromatic derivatives have attracting theoretical interest from the viewpoint of their aromaticity. Cyclopent[*a*]azulene, which consists of cyclopentadiene and azulene fused together is one of the promising precursors for the azulene derivatives with extended π-conjugation. Therefore, Yasunami et al. attempted to prepare cyclopent[*a*]azulenes **136** and **137** and elucidated their reactivity (Scheme 33) [62]. The reaction of **131a** with *N*-bromosuccinimide (NBS) in carbon tetrachloride at 0 °C yields monobromide **132**. Attempts of the elimination of hydrogen bromide from **132** with amine are failed to obtain the desired elimination product **133**. However, treatment of **132** in refluxing chloroform converts to **133** in 87% yield (two-step yield from **131a**). Treatment of **133** with 100% H_3_PO_4_ does not afford the desired products, but forms unidentifiable compounds. Hence, first, Diels–Alder reaction with cyclopentadiene is employed to **133** to produce **134**. Following hydrolysis of the ester group and acid-catalyzed decarboxylation result in **135**. When **135** is treated under FVP conditions (400 °C, 0.5–0.05 mmHg), **136** and **137** are obtained almost quantitatively (96%) in a 1:1 ratio.

**Scheme 34:**
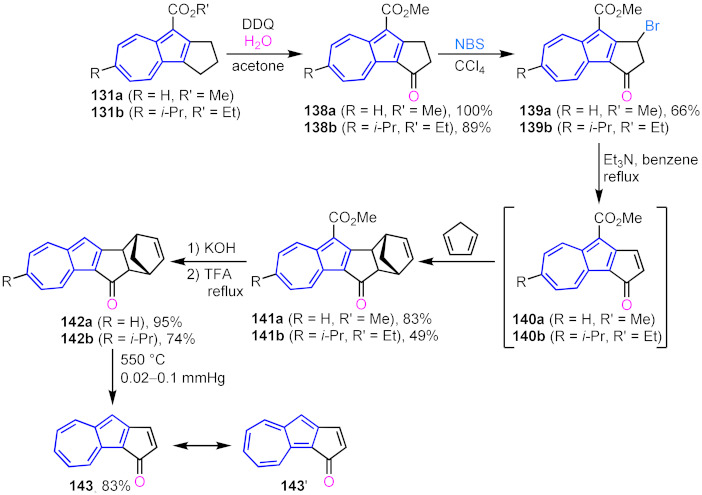
Synthesis of 3*H*-cyclopent[*a*]azulen-3-one **143** and its precursors.

**Scheme 35:**
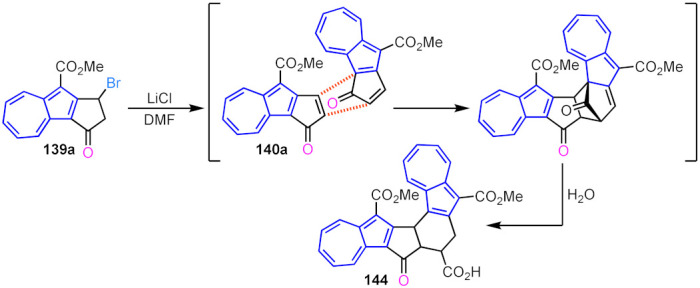
Formation of **144** by the Diels–Alder type cyclodimerization of **140a**.

Oxidation of dihydrocyclopent[*a*]azulenes **131a** and **131b** with DDQ in an aqueous acetone solution produces the ketones **138a** and **138b** in excellent yields, which are subsequently converted to the monobromides **139a** and **139b** by the bromination with NBS (Scheme 34) [63]. The elimination of hydrogen bromide from **139a** and **139b** by the treatment with triethylamine generates 3*H*-cyclopent[*a*]azulen-3-ones **140a** and **140b** as unstable intermediates, which are trapped by cyclopentadiene to give the bridged compounds **141a** and **141b**. In the absence of cyclopentadiene, the product **140a** generated by the elimination reaction from **139a** undergoes a Diels–Alder type cyclodimerization reaction to produce **144** (Scheme 35). The products **142a** and **142b** can be prepared by trifluoroacetic acid-catalyzed decarboxylation of the carboxylic acids obtained by the hydrolysis of **141a** and **141b**, although these compounds undergo decomposition when they are treated with 100% H_3_PO_4_. The compound **142a** can be sublimed by the FVP at 550 °C to afford 3*H*-cyclopent[*a*]azulen-3-one **143**.

It is well known that the ring-fused azulene derivatives exhibit a bond-length alternation in the seven-membered ring, which is reflected in the vicinal coupling constants in their ^1^H NMR spectra [64,65,66]. In the ^1^H NMR spectrum of **143**, the bond-length alternation is smaller than that of the usual ring-fused azulene derivatives, because the contribution of the resonance structure of **143’** is more significant to avoid the unstable anti-aromatic cyclopentadienone substructure in **143**.

**Scheme 36:**
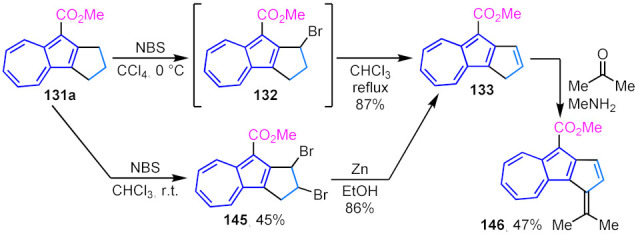
Synthesis of dimethylfulvene-fused azulene derivative **146**.

Bromination of **131a** with NBS yields unstable bromide **132**, which can be used in subsequent reactions without further purification. HBr is readily eliminated from **132** in chloroform under the reflux condition to give **133** as also described in Scheme 33. The product **133** is also prepared by the bromination of **131a** with NBS in chloroform at room temperature to afford the dibromide **145** in 45% yield, followed by the treatment with zinc powder in ethanol to afford **133** in 86% yield (Scheme 36). The methylene position of the cyclopentadiene moiety of **133** is readily deprotonated upon the treatment with amines, and subsequent condensation reaction with ketones and aldehydes gives pentafulvene-fused azulene derivatives. For example, the reaction of **133** with acetone in the presence of methylamine furnishes **146** in 47% yield. Condensation reactions of **133** with carbonyl compounds other than acetone are also investigated to produce the desired pentafulvene derivatives with moderate to good yields.

Spectroscopic properties and bond-length alternations of pentafulvene **146** and its derivatives obtained by this procedure are evaluated by UV/Vis spectra, as well as by ^1^H NMR spectra.

**Scheme 37:**
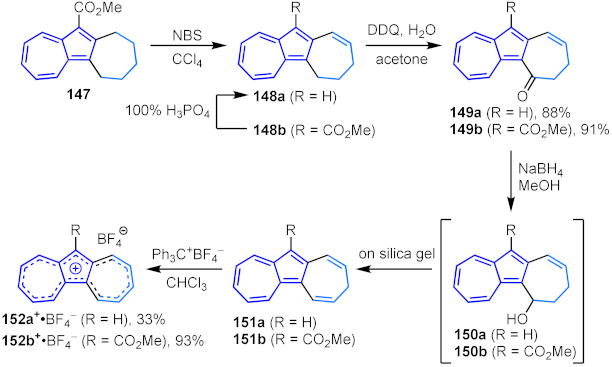
Synthesis of cyclohept[*a*]azulenylium ions **152a^+^** and **152b^+^**.

Cyclohept[*a*]azulenylium ion is one of the non-benzene aromatic compounds with a tricyclic carbon skeleton. The theoretical calculations suggest that this ion is a stable cation with a contribution of a 14 π-electron system. Yasunami et al. have investigated the synthesis of cyclohept[*a*]azulenylium ions **152a^+^** and **152b^+^** in order to demonstrate its stability and properties (Scheme 37) [67].

The reaction of the ester derivative **147** with 1.2 equivalents of NBS in refluxing CCl_4_ gives olefinic compound **148b** in 85% yield. The decarboxylation of **148b** with 100% H_3_PO_4_ affords **148a**. Treatment of **148a** and **148b** with 2.2 equivalents of DDQ in aqueous acetone solution resulted in the carbonyl derivatives **149a** and **149b** in 88 and 91% yields, respectively. Reduction of **149a** and **149b** with sodium borohydride (NaBH_4_) leads to unstable alcohols **150a** and **150b**, which are easily dehydrated by passing through a silica gel column yielding 3*H*-cyclohept[*a*]azulenes **151a** and **151b**. Treatment of **151a** and **151b** in chloroform with Ph_3_C^+^BF_4_^−^ provides cyclohept[*a*]azulenylium ions **152a^+^** (33%) and **52b^+^** (93%) as tetrafluoroborates.

The ^1^H NMR spectra of **152a^+^** and **152b^+^** show the downfield shift of the ring proton signals attributed to their ionic structures, as well as the observation as equivalent proton signals at the two seven-membered rings. Furthermore, the fact that the ^13^C NMR spectra of **152a^+^** and **152b^+^** exhibit only eight signals corresponding to the ring carbons suggesting the delocalization of the positive charge of **152a^+^** and **152b^+^** in both seven-membered rings.

**Scheme 38:**
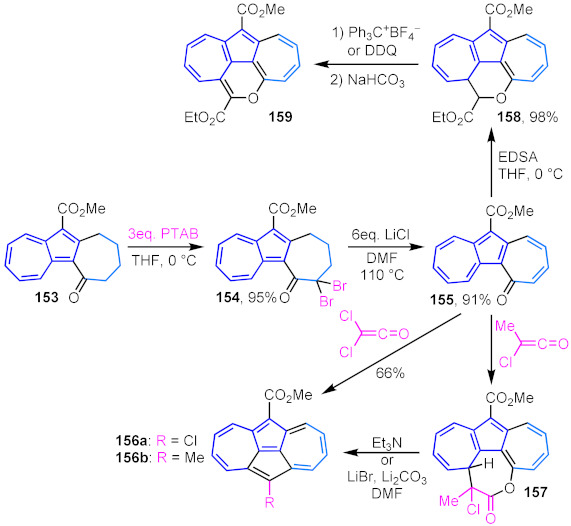
Synthesis of dicyclohepta[*cd*,*gh*]pentalenes **156a**,**b** and 5-oxa-5*H*-dicyclohept[*cd*,*hi*]indene **159**.

Dicyclohepta[*cd*,*gh*]pentalenes have been focused as one of the bridging [14] annulenes and the synthesis was achieved by Vogel and Reel in 1972 [68]. Inspired by their report, Yasunami and co-workers developed a novel approach for the synthesis of dicyclohepta[*cd*,*gh*]pentalenes **156a** and **156b** by the cyclization of 5*H*-cyclohept[*a*]azulen-5-one **155** with haloketenes (Scheme 38) [69]. The synthetic precursor **155** was prepared by a two-step route using **153** as a starting material; **153** is brominated with three equivalents of phenyltrimethylammonium perbromide (PTAB) at 0 °C to give dibromo derivative **154** in 95% yield, followed by debromination with six equivalents of lithium chloride (LiCl) in *N*,*N*-dimethylformamide (DMF) at 110 °C under a nitrogen atmosphere to form **155** in 91% yield. The reaction of **155** with dichloroketene for 11 hours in benzene under the reflux condition produces dicyclohepta[*cd*,*gh*]pentalene **156a** in 66% yield. On the other hand, the reaction of **155** with chloromethyl ketene under the similar conditions gives the lactone derivative **157** within five minutes. The conversion of lactone **157** to dicyclohepta[*cd*,*gh*]pentalene **156b** can be achieved by heating in triethylamine or in dimethylformamide at 120 °C with lithium bromide and lithium carbonate.

The reaction of **155** with sulfur ylide, i.e., ethyl dimethyl sulfinylidene acetate (EDSA), produces **158** in 98% yield (Scheme 38) [70]. Oxidation of **158** with Ph_3_C^+^BF_4_^−^ produces a cationic intermediate, which can be treated with a sodium bicarbonate solution to furnish 5-oxa-5*H*-dicyclohept[*cd*,*hi*]indene **159** in 24% yield. Treatment of **158** with DDQ instead of Ph_3_C^+^BF_4_^−^ provides **159** in a two-step yield of 65%.

**Scheme 39:**
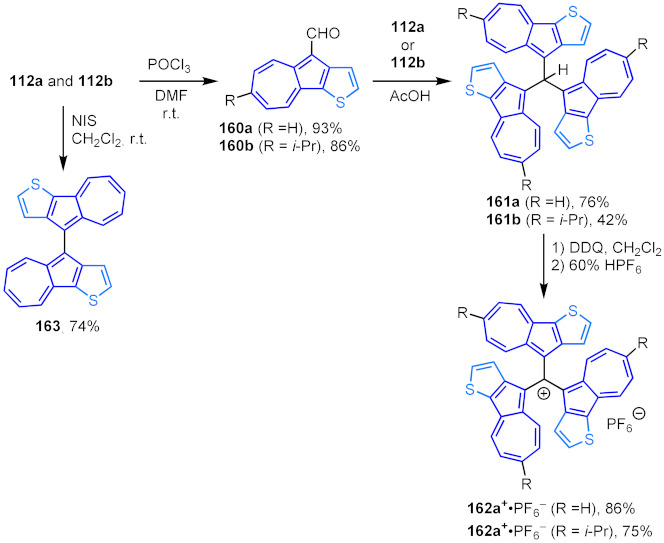
Synthesis of tri(1-azulenyl)methylium ions **162a^+^** and **162b^+^** and 1,1’-biazulene derivative **163** by the reaction of azuleno[1,2-*b*]thiophenes **112a** and **112b**.

Ito et al. reported the first synthesis of tri(1-azulenyl)methylium ions **162a^+^** and **162b^+^** from azuleno[1,2-*b*]thiophenes **112a** and **112b** and revealed their bond-length alternations by both of the coupling constants in ^1^H NMR spectra and single-crystal X-ray structure analysis (Scheme 39) [59]. The Vilsmeier reaction of **112a** and **112b** affords formyl derivatives **160a** (93%) and **160b** (86%), which are condensed with two equivalents of **112a** or **112b** in acetic acid at room temperature to give the corresponding tri(1-azulenyl)methanes **161a** and **161b** in 76 and 42% yields, respectively. Hydride abstraction reaction of **161a** and **161b** with DDQ and subsequent anion exchange with 60% HPF_6_ solution provides tris(azuleno[1,2-*b*]thiophene-9-yl)methylium ions **162a^+^** and **162b^+^** as hexafluorophosphates in 86 and 75% yields, respectively. In ^1^H NMR, the vicinal coupling constants in the seven-membered ring of **162a^+^** show the alternating pattern of *J* = 8.8 and 10.5 Hz, indicating a clear contribution of the bond-length alternation. In the 6-isopropyl derivative **162b^+^**, the coupling constant increases slightly compared to those of **162a^+^**. The results of X-ray crystallography show that the azuleno[1,2-*b*]thiophene moiety of the carbocation derived from the 6-isopropyl derivative has an almost planar structure and distinct difference in the carbon–carbon bond length of the seven-membered ring, as expected from the ^1^H NMR spectrum.

Azulene derivatives usually react with NIS to give the corresponding 1-iodoazulene derivatives [71]. However, in the reaction of **112a** with NIS, 1,1’-biazulene derivative **163** is formed in 74% yield instead of the expected iodoazulene derivative (Scheme 39) [72]. A similar reaction with NBS and NCS forms neither the corresponding 1-haloazulenes or the 1,1’-biazulene derivative **163**, but only the decomposition is observed. Therefore, this homocoupling reaction is a specific reactivity between **112a** and NIS, and a slight difference in the oxidation ability of NXS should be attributed to the outcome of the reaction.

**Scheme 40:**
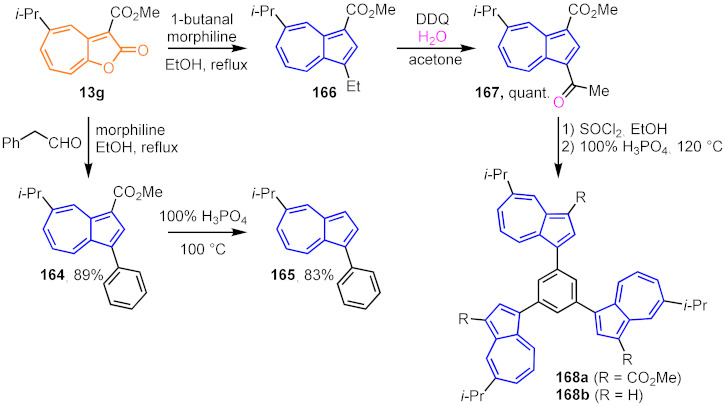
Synthesis of 1-phenylazulenes **164** and **165**, and 1,3,5-tri(1-azulenyl)benzenes **168a** and **168b** via the reaction of 2*H*-cyclohepta[*b*]furan-2-one **13g** with *in situ* generated enamines.

1-Phenylazulene and 1,3,5-tri(1-azulenyl)benzenes can be prepared from 5-isopropyl-2*H*-cyclohepta[*b*]furan-2-one **13g** (Scheme 40). The [8 + 2] cycloaddition reaction of **13g** with *in situ* generated enamine from phenylacetaldehyde and morpholine gives 1-phenylazulene **164** having a ester function in 89% yield [73]. Removal of the ester function of **164** is accomplished by heating in 100% H_3_PO_4_ to produce **165** in 83% yield. The synthesis of 1,3,5-tri(1-azulenyl)benzenes **168a** and **168b** is also performed in four steps using **13g** as a starting material. The 1-acetylazulene derivative **167** is obtained by the reaction of **13g** with enamine prepared from 1-butanal and morpholine, followed by the oxidation of the α-position of azulene ring by DDQ in aqueous acetone furnishes **167**, quantitatively. Benzannulation of **167** with thionyl chloride (SOCl_2_) in ethanol produces 1,3,5-(1-azulenyl)benzene **168a** in 56% yield. 1,3,5-Tris(1-azulenyl)benzene **168a** is thought to be generated by trimerization-type benzannulation by successive aldol condensation of the acetyl group of **167** [74]. The ester group of **168a** is also decarboxylated by 100% H_3_PO_4_ to provide **168b** in 98% yield. The 3-position of the azulene ring in **165** and **168** can be functionalized by electrophilic substitution reactions.

## 7. Synthesis of 2-arylazulenes by the Reaction of 2*H*-cyclohepta[*b*]furan-2-ones with Silyl Enol Ethers

**Scheme 41:**
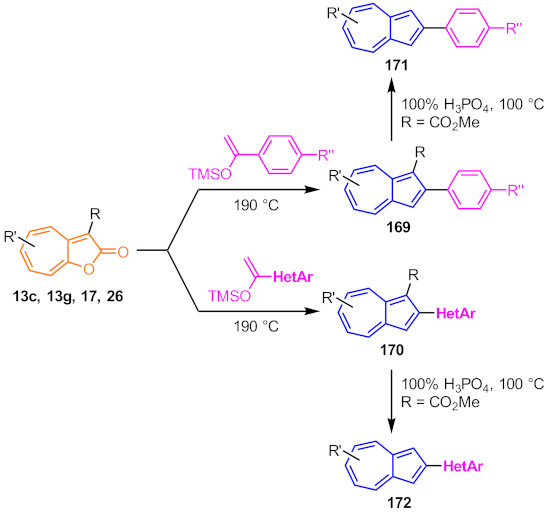
Synthesis of 2-arylazulenes 169–172 by the reaction of 2*H*-cyclohepta[*b*]furan-2-ones with aryl-substituted silyl enol ethers.

The introduction of aryl moiety to the 2-position of azulene ring is mostly achieved by the cross-coupling reactions using the corresponding haloazulenes as starting materials because of the low reactivity of electrophiles to these sites [75,76,77,78]. There is only one example of the electrophilic substitution reaction by heterocyclic compound at the 2-position of the azulene ring, but in this case, a strong electron-donating group such as dimethylamino group at the 6-position and protection at the 1,3-positions of the azulene ring are both essential [79,80]. Recently, the reaction of 2*H*-cyclohepta[*b*]furan-2-ones **13c**, **13g**, **17**, and **26** with silyl enol ethers substituted by various aryl groups have been reported as a new synthetic method for 2-arylazulenes **169**–**172** (Scheme 41) [81]. This method is applied to the synthesis of 2-arylazulenes, such as 2-(phenyl-, naphthyl-, ferrocenyl-, and heteroaryl)azulenes, although the reaction requires high temperature (190 °C). This method provides 2-arylazulenes, which are difficult to prepare by the reaction using enamines, in good to excellent yields. Furthermore, the ester group of **169** and **170** can be removed by decarboxylation with 100% H_3_PO_4_ to give the parent derivatives **171** and **172** in excellent yields. The reaction mechanism for the formation of 2-arylazulenes is thought to be via an [8 + 2] cycloaddition reaction between 2*H*-cyclohepta[*b*]furan-2-ones and silyl enol ethers, similar to the reaction with enamines.

**Figure 3:**
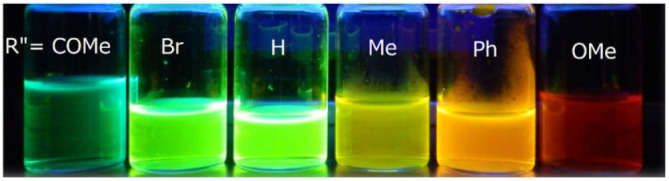
Photos of 2-phenylazulenes 171 in acidic media (30% CF_3_CO_2_H/CH_2_Cl_2_) under the irradiation of UV-light (λ_ex_ = 365 nm).

The 2-arylazulenes **171** do not show any luminescence in neutral media (i.e., in dichloromethane), whereas they exhibit pronounced fluorescence when trifluoroacetic acid is added to the solution (Figure 3). The emission maxima of **171** in the acidic solution depend on the electronic nature of the substituent at the p-position of the benzene ring. The compounds with an electron-donating group at the p-position of the benzene ring show the red-shift in the luminescence, while the derivatives with an electron-withdrawing group exhibit the opposite effect, i.e., blue-shift.

## 8. Conclusions

In this review, we summarize the preparation of 2*H*-cyclohepta[*b*]furan-2-ones and their conversion to azulene derivatives, as well as their reactivities and properties. The reaction of 2*H*-cyclohepta[*b*]furan-2-ones with active methylenes, olefins, enamines, and silyl enol ethers can lead to azulene derivatives, and each of these synthetic methods has its individual advantages.

The reaction of 2*H*-cyclohepta[*b*]furan-2-ones with active methylenes provides 2-amino- and 2-hydroxyazulene derivatives, similar to the azulene synthesis from tropone derivatives reported by Nozoe et al. Furthermore, the amino and hydroxy groups of these derivatives can be further converted into a variety of functional groups. 2*H*-Cyclohepta[*b*]furan-2-ones react with olefins and their analogues to generate multiply functionalized azulene derivatives, such as ring-fused, alcohol, and alkyl-substituted ones. The most frequently employed method for the synthesis of azulenes starting from 2*H*-cyclohepta[*b*]furan-2-ones is the procedure reported by Yasunami-Takase et al. employing enamines, in which ring-fusing, alkyl, and aryl substituted derivatives can be prepared. However, unfortunately, this method is not suitable for the synthesis of 2-arylazulenes owing to the low yields. In contrast, the reaction of 2*H*-cyclohepta[*b*]furan-2-ones with aryl-substituted silyl enol ethers can circumvent such problems, and the corresponding 2-arylazulenes (including heteroaryls) are obtained in good to excellent yields.

Since the methodologies described above are extremely effective for the preparation of novel azulene derivatives with potential as organic materials and pharmaceuticals, we hope that this review will contribute to the development of these fields.

## Figures and Tables

**Table 1:** Reaction of 2*H*-cyclohepta[*b*]furan-2-ones with active methylenes.

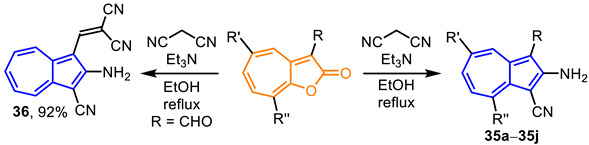				
Entry	R	R’	R’’	Product, Yield [%]
1	CO_2_Me	H	H	**35a**, 91
2	CO_2_Me	*i-*Pr	H	**35b**, 93
3	SO_2_Me	H	H	**35c**, 87
4	SO_2_Me	*i-*Pr	H	**35d**, 89
5	S^+^Me_2_	H	H	**35e**, 90
6	SMe	H	H	No reaction
7	I	*i-*Pr	H	No reaction
8	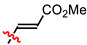	*i-*Pr	H	**35f**, 88
9	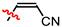	*i-*Pr	H	**35g**, 90
10	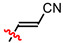	*i-*Pr	H	**35h**, 88
11	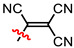	*i-*Pr	H	**35i**, 92
12	CO_2_Et	H	OMe	**35j**, 85

**Table 2:** The reaction of **13c** with 6,6-dimethylfulvene.

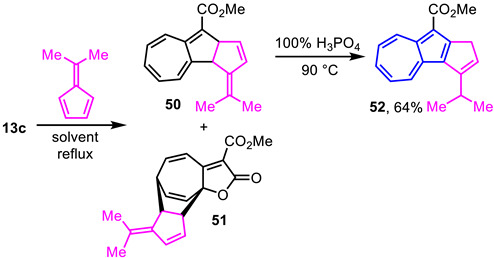			
Entry	Solvent	50, Yield [%]	51, Yield [%]
1	xylene	35	0
2	benzene	21	28
3	ethanol	17	44

**Table 3:**
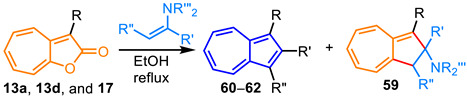
Synthesis of azulene derivatives **55**–**57** by the [8 + 2] cycloaddition of 2*H*-cyclohepta[*b*]furan-2-ones with various enamines.

Entry	Substrate	R	Carbonyl compound	Amine	Product, Yield [%]^2^
1	**17**	H	propanal	pyrrolidine	**60a**, 26
2	**17**	H	butanal	pyrrolidine	**60b**, 90
3	**17**	H	3-methylbutanal	pyrrolidine	**60c**, 72
4	**17**	H	valeraldehyde	pyrrolidine	**60d**, 64
5	**17**	H	phenylacetoaldehyde	pyrrolidine	**60e**, 41
6	**17**	H	2-butanone	pyrrolidine	77 (**60f**: **60g** = 1:1)
7	**17**	H	3-pentanone	pyrrolidine	**60h**, 83
8	**17**	H	acetophenone	pyrrolidine	**60i**, 29
9	**17**	H	cyclopentanone	pyrrolidine	**60j**, 10 (**59**, 83) ^1^
10	**17**	H	cyclohexanone	pyrrolidine	**60k**, 95
11	**17**	H	cycloheptanone	pyrrolidine	**60l**, 95
12	**17**	H	cyclooctanone	pyrrolidine	**60m**, 81
13	**17**	H	cyclodecanone	pyrrolidine	**60n**, 41
14	**17**	H	butanal	morpholine	**60b**, 26
15	**17**	H	3-methylbutanal	morpholine	**60c**, 18
16	**17**	H	2-butanone	morpholine	28 (**60f**: **60g** = 1:1)
17	**17**	H	3-pentanone	morpholine	**60h**, 19
18	**17**	H	cyclopentanone	morpholine	**60j**, 44
19	**17**	H	cyclohexanone	morpholine	**60k**, 13
20	**17**	H	cycloheptanone	morpholine	**60l**, 76
21	**13a**	CO_2_Et	cyclopentanone	pyrrolidine	**61a**, 17
22	**13a**	CO_2_Et	cyclohexanone	pyrrolidine	**61b**, 2
23	**13a**	CO_2_Et	cycloheptanone	pyrrolidine	**61c**, trace
24	**13a**	CO_2_Et	butanal	morpholine	**61d**, 88
25	**13a**	CO_2_Et	cyclopentanone	morpholine	**61a**, 88
26	**13a**	CO_2_Et	cyclohexanone	morpholine	**61b**, trace
27	**13a**	CO_2_Et	cycloheptanone	morpholine	**61c**, 5
28	**13d**	CN	cyclopentanone	pyrrolidine	**62a,** 5 (**59**, 81) ^1^
29	**13d**	CN	cyclohexanone	pyrrolidine	**62b,** 13
30	**13d**	CN	cycloheptanone	pyrrolidine	**62c,** 25
31	**13d**	CN	butanal	pyrrolidine	**62d**, 23
32	**13d**	CN	cyclopentanone	morpholine	**62a,** 2 (**59**, 97) ^1^
33	**13d**	CN	cyclohexanone	morpholine	**62b,** 44
34	**13d**	CN	cycloheptanone	morpholine	**62c,** 86
35	**13d**	CN	butanal	morpholine	**62d**, 12 (**59**, 75) ^1^

^1^ The value in the parentheses is the isolated yield of aminohydroazulene intermediates **59**. ^2^ The reaction is carried out with a unified reaction time of about 2 hours.
